# (2*E*)-1-(5-Chloro­thio­phen-2-yl)-3-(2,4,5-trimeth­oxy­phen­yl)prop-2-en-1-one

**DOI:** 10.1107/S1600536811028236

**Published:** 2011-07-23

**Authors:** A. N. Prabhu, A. Jayarama, T. N. Guru Row, V. Upadhyaya

**Affiliations:** a Physics Department, Manipal Institute of Technology, Manipal University, Manipal 576 104, India; b Department of Physics, Mangalore Institute of Technology & Engineering (MITE), Badagamijar, Moodabidri, Karnataka, India; c Solid State and Structural Chemistry Unit, Indian Institute of Science, Bangalore, India

## Abstract

In the title mol­ecule, C_16_H_15_ClO_4_S, the chloro­thio­phene and trimeth­oxy­phenyl rings make a dihedral angle of 31.12 (5)°. The C=C double bond exhibits an *E* conformation. In the crystal, C—H⋯O inter­actions generate bifurcated bonds, linking the mol­ecules into chains along the *b* axis.

## Related literature

For general background to chalcones, see: Tomazela *et al.* (2000 ▶); Uchida *et al.* (1998 ▶); Zyss & Chemla (1987 ▶). For related structures, see: Benmekhbi *et al.* (2009 ▶).
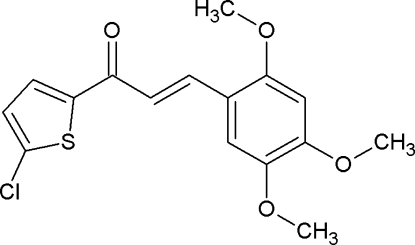


## Experimental

### 

#### Crystal data

C_16_H_15_ClO_4_S
*M* *_r_* = 338.79
Monoclinic, 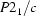

*a* = 18.2795 (12) Å
*b* = 9.0393 (7) Å
*c* = 9.8673 (6) Å
β = 99.390 (4)°
*V* = 1608.57 (19) Å^3^
*Z* = 4
Mo *K*α radiation
μ = 0.38 mm^−1^
*T* = 296 K
0.20 × 0.18 × 0.18 mm

#### Data collection

Bruker SMART APEX CCD detector diffractometer
Absorption correction: multi-scan (*SADABS*; Bruker, 1998 ▶) *T*_min_ = 0.928, *T*_max_ = 0.935
11590 measured reflections
4590 independent reflections
1621 reflections with *I* > 2σ(*I*)
*R*_int_ = 0.065

#### Refinement

*R*[*F* ^2^ > 2σ(*F* ^2^)] = 0.064
*wR*(*F* ^2^) = 0.197
*S* = 0.94
4590 reflections
202 parameters
H-atom parameters constrained
Δρ_max_ = 0.39 e Å^−3^
Δρ_min_ = −0.32 e Å^−3^

### 

Data collection: *SMART* (Bruker, 1998 ▶); cell refinement: *SAINT-Plus* (Bruker, 1998 ▶); data reduction: *SAINT-Plus*; program(s) used to solve structure: *SHELXS97* (Sheldrick, 2008 ▶); program(s) used to refine structure: *SHELXL97* (Sheldrick, 2008 ▶); molecular graphics: *ORTEP-3* (Farrugia, 1999 ▶); software used to prepare material for publication: *WinGX* (Farrugia, 1999 ▶).

## Supplementary Material

Crystal structure: contains datablock(s) global, I. DOI: 10.1107/S1600536811028236/pv2418sup1.cif

Structure factors: contains datablock(s) I. DOI: 10.1107/S1600536811028236/pv2418Isup2.hkl

Supplementary material file. DOI: 10.1107/S1600536811028236/pv2418Isup3.cml

Additional supplementary materials:  crystallographic information; 3D view; checkCIF report

## Figures and Tables

**Table 1:** Hydrogen-bond geometry (Å, °)

*D*—H⋯*A*	*D*—H	H⋯*A*	*D*⋯*A*	*D*—H⋯*A*
C3—H3⋯O1^i^	0.93	2.71	3.476 (4)	140
C6—H6⋯O1^i^	0.93	2.68	3.597 (5)	169

Symmetry code: (i) 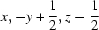.

**Fig. 1.**
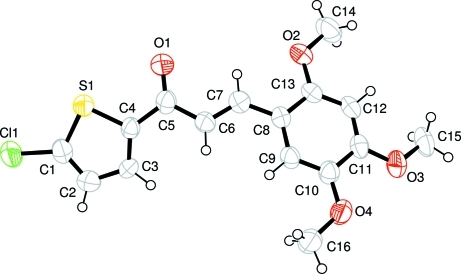
ORTEP (Farrugia, 1999) view of the title compound, showing 50% probability ellipsoids and the atom numbering scheme.

**Fig. 2.**
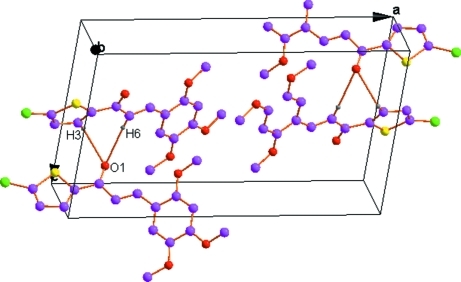
A unit cell packing of the title compound showing intermolecular interactions with dotted lines. H-atoms not involved in hydrogen bonding have been excluded.

## supplementary crystallographic information

### Comment

Chalcones represent one of the most abundant and ubiquitous group of natural products (Tomazela *et al.*, 2000). Recently, it has been noted that, among many organic compounds reported for their second harmonic generation, chalcone derivatives are known for their excellent blue light transmittance and good crystallizability (Uchida *et al.*, 1998). The title compound is found to be of interest as an organic non-linear optical material (Zyss & Chemla, 1987).

In the title molecule (Fig. 1), the chlorothiophene and trimethoxyphenyl rings are non-planar with a dihedral angle of 31.12 (5)°. The C═C double bond exhibits an *E* conformation. In the crystal structure, C—H···O interaction generates  H-bonds from two donors, C3 and C6 to the same acceptor, O1 linking the molecules into chains along the *c*-axis (Fig 2).

### Experimental

To synthesize the title compound, commercially available Analytical Reagent (AR)-grade chemicals were used. 2-Acetyl-5-chlorothiophene (0.01 mol) and 2,4,5-trimethoxybenzaldehyde (0.01 mol) were dissolved in methanol (60 ml). Sodium hydroxide (5 ml, 20%) was then added drop wise to the solution, and stirred for 2 h. The content of the flask were poured into ice-cold water, and the resulting crude solid was collected by filtration. The compound was dried in a hot-air-oven at 323 K and re-crystallized twice from acetone.

### Refinement

The H atoms were placed at calculated positions in the riding model approximation with C—H = 0.93 and 0.96 Å for aryl and methyl type H-atoms, respectively, and *U*_iso_(H) = 1.2 or 1.5*U*_eq_(C).

### Crystal data

**Table:**

C_16_H_15_ClO_4_S	*F*(000) = 704
*M**_r_* = 338.79	*D*_x_ = 1.399 Mg m^−^^3^
Monoclinic, *P*2_1_/*c*	Mo *K*α radiation, λ = 0.71073 Å
Hall symbol: -P 2ybc	Cell parameters from 4590 reflections
*a* = 18.2795 (12) Å	θ = 2.3–30.5°
*b* = 9.0393 (7) Å	µ = 0.38 mm^−^^1^
*c* = 9.8673 (6) Å	*T* = 296 K
β = 99.390 (4)°	Block, yellow
*V* = 1608.57 (19) Å^3^	0.20 × 0.18 × 0.18 mm
*Z* = 4	

### Data collection

**Table:**

Bruker SMART APEX CCD detector diffractometer	4590 independent reflections
Radiation source: fine-focus sealed tube	1621 reflections with *I* > 2σ(*I*)
graphite	*R*_int_ = 0.065
ω scans	θ_max_ = 30.5°, θ_min_ = 2.3°
Absorption correction: multi-scan (*SADABS*; Bruker, 1998)	*h* = −26→26
*T*_min_ = 0.928, *T*_max_ = 0.935	*k* = −12→12
11590 measured reflections	*l* = −13→11

### Refinement

**Table:**

Refinement on *F*^2^	Primary atom site location: structure-invariant direct methods
Least-squares matrix: full	Secondary atom site location: difference Fourier map
*R*[*F*^2^ > 2σ(*F*^2^)] = 0.064	Hydrogen site location: inferred from neighbouring sites
*wR*(*F*^2^) = 0.197	H-atom parameters constrained
*S* = 0.94	*w* = 1/[σ^2^(*F*_o_^2^) + (0.0776*P*)^2^] where *P* = (*F*_o_^2^ + 2*F*_c_^2^)/3
4590 reflections	(Δ/σ)_max_ = 0.001
202 parameters	Δρ_max_ = 0.39 e Å^−^^3^
0 restraints	Δρ_min_ = −0.32 e Å^−^^3^

### Special details

**Table:**

Geometry. All s.u.'s (except the s.u. in the dihedral angle between two l.s. planes) are estimated using the full covariance matrix. The cell s.u.'s are taken into account individually in the estimation of s.u.'s in distances, angles and torsion angles; correlations between s.u.'s in cell parameters are only used when they are defined by crystal symmetry. An approximate (isotropic) treatment of cell s.u.'s is used for estimating s.u.'s involving l.s. planes.
Refinement. Refinement of *F*^2^ against ALL reflections. The weighted *R*-factor *wR* and goodness of fit *S* are based on *F*^2^, conventional *R*-factors *R* are based on *F*, with *F* set to zero for negative *F*^2^. The threshold expression of *F*^2^ > 2σ(*F*^2^) is used only for calculating *R*-factors(gt) *etc*. and is not relevant to the choice of reflections for refinement. *R*-factors based on *F*^2^ are statistically about twice as large as those based on *F*, and *R*- factors based on ALL data will be even larger.

### Fractional atomic coordinates and isotropic or equivalent isotropic displacement parameters (Å^2^)

**Table:**

	*x*	*y*	*z*	*U*_iso_*/*U*_eq_	
Cl1	1.19089 (6)	0.43556 (14)	0.13180 (12)	0.0853 (4)	
S1	1.03627 (5)	0.43686 (11)	0.18651 (10)	0.0602 (3)	
O1	0.88221 (14)	0.3967 (3)	0.2335 (3)	0.0681 (8)	
O2	0.66429 (13)	0.1207 (3)	0.2390 (3)	0.0726 (8)	
O3	0.55841 (13)	−0.2151 (3)	−0.1240 (3)	0.0709 (8)	
O4	0.66030 (14)	−0.1426 (3)	−0.2658 (3)	0.0810 (9)	
C1	1.10339 (19)	0.3619 (4)	0.1052 (4)	0.0596 (10)	
C2	1.0792 (2)	0.2480 (5)	0.0231 (4)	0.0704 (12)	
H2	1.1088	0.1949	−0.0279	0.085*	
C3	1.00321 (19)	0.2181 (4)	0.0233 (4)	0.0547 (9)	
H3	0.9770	0.1434	−0.0282	0.066*	
C4	0.97267 (17)	0.3111 (4)	0.1075 (3)	0.0489 (9)	
C5	0.89668 (19)	0.3155 (4)	0.1409 (4)	0.0553 (10)	
C6	0.84197 (18)	0.2173 (4)	0.0627 (4)	0.0539 (9)	
H6	0.8521	0.1744	−0.0179	0.065*	
C7	0.77806 (18)	0.1879 (4)	0.1045 (4)	0.0537 (9)	
H7	0.7700	0.2364	0.1839	0.064*	
C8	0.71877 (17)	0.0894 (4)	0.0419 (4)	0.0474 (9)	
C9	0.71853 (19)	0.0245 (4)	−0.0852 (4)	0.0520 (9)	
H9	0.7561	0.0484	−0.1348	0.062*	
C10	0.66447 (19)	−0.0742 (4)	−0.1407 (4)	0.0555 (10)	
C11	0.60855 (18)	−0.1124 (4)	−0.0638 (4)	0.0535 (10)	
C12	0.60767 (18)	−0.0500 (4)	0.0619 (4)	0.0541 (10)	
H12	0.5708	−0.0757	0.1123	0.065*	
C13	0.66189 (18)	0.0522 (4)	0.1149 (4)	0.0516 (9)	
C14	0.6146 (2)	0.0767 (5)	0.3268 (4)	0.0876 (15)	
H14A	0.5646	0.0937	0.2821	0.131*	
H14C	0.6240	0.1331	0.4102	0.131*	
H14B	0.6213	−0.0266	0.3477	0.131*	
C15	0.5106 (2)	−0.2793 (4)	−0.0396 (5)	0.0771 (13)	
H15C	0.5398	−0.3263	0.0383	0.116*	
H15A	0.4792	−0.3515	−0.0916	0.116*	
H15B	0.4805	−0.2034	−0.0084	0.116*	
C16	0.7194 (2)	−0.1165 (5)	−0.3418 (4)	0.0914 (16)	
H16B	0.7202	−0.0137	−0.3658	0.137*	
H16C	0.7118	−0.1753	−0.4239	0.137*	
H16A	0.7657	−0.1430	−0.2866	0.137*	

### Atomic displacement parameters (Å^2^)

**Table:**

	*U*^11^	*U*^22^	*U*^33^	*U*^12^	*U*^13^	*U*^23^
Cl1	0.0596 (6)	0.1155 (10)	0.0833 (9)	−0.0246 (6)	0.0194 (5)	−0.0174 (7)
S1	0.0600 (6)	0.0680 (7)	0.0544 (7)	−0.0145 (5)	0.0151 (4)	−0.0090 (5)
O1	0.0639 (16)	0.083 (2)	0.0595 (19)	−0.0111 (14)	0.0169 (13)	−0.0174 (16)
O2	0.0636 (17)	0.089 (2)	0.074 (2)	−0.0108 (14)	0.0351 (14)	−0.0162 (17)
O3	0.0568 (15)	0.0707 (18)	0.087 (2)	−0.0218 (14)	0.0168 (14)	−0.0001 (15)
O4	0.0796 (19)	0.103 (2)	0.063 (2)	−0.0404 (17)	0.0187 (15)	−0.0202 (17)
C1	0.052 (2)	0.070 (3)	0.057 (3)	−0.008 (2)	0.0081 (17)	0.007 (2)
C2	0.063 (2)	0.079 (3)	0.072 (3)	0.000 (2)	0.019 (2)	−0.017 (2)
C3	0.053 (2)	0.052 (2)	0.059 (3)	−0.0082 (18)	0.0088 (17)	−0.018 (2)
C4	0.051 (2)	0.054 (2)	0.042 (2)	−0.0120 (17)	0.0071 (16)	0.0042 (18)
C5	0.058 (2)	0.060 (2)	0.047 (3)	−0.0123 (19)	0.0077 (17)	0.009 (2)
C6	0.053 (2)	0.064 (2)	0.047 (2)	−0.0090 (18)	0.0144 (17)	−0.0016 (19)
C7	0.055 (2)	0.055 (2)	0.052 (2)	0.0010 (18)	0.0088 (17)	0.0050 (19)
C8	0.0426 (18)	0.048 (2)	0.052 (2)	−0.0011 (16)	0.0083 (16)	0.0051 (18)
C9	0.050 (2)	0.062 (2)	0.046 (2)	−0.0100 (18)	0.0128 (16)	0.0075 (19)
C10	0.054 (2)	0.060 (2)	0.052 (2)	−0.0082 (19)	0.0065 (17)	0.004 (2)
C11	0.0377 (18)	0.052 (2)	0.070 (3)	0.0009 (16)	0.0071 (17)	0.007 (2)
C12	0.0421 (19)	0.056 (2)	0.068 (3)	0.0032 (17)	0.0200 (17)	0.007 (2)
C13	0.0451 (19)	0.055 (2)	0.057 (3)	0.0070 (18)	0.0159 (16)	0.002 (2)
C14	0.073 (3)	0.127 (4)	0.070 (3)	−0.002 (3)	0.032 (2)	0.000 (3)
C15	0.049 (2)	0.065 (3)	0.121 (4)	−0.011 (2)	0.024 (2)	0.010 (3)
C16	0.102 (3)	0.118 (4)	0.062 (3)	−0.052 (3)	0.033 (2)	−0.021 (3)

### Geometric parameters (Å, °)

**Table:**

Cl1—C1	1.713 (4)	C7—C8	1.460 (5)
S1—C1	1.711 (4)	C7—H7	0.9300
S1—C4	1.719 (3)	C8—C9	1.384 (5)
O1—C5	1.234 (4)	C8—C13	1.399 (4)
O2—C13	1.367 (4)	C9—C10	1.377 (5)
O2—C14	1.411 (4)	C9—H9	0.9300
O3—C11	1.370 (4)	C10—C11	1.412 (4)
O3—C15	1.426 (4)	C11—C12	1.365 (5)
O4—C10	1.372 (4)	C12—C13	1.393 (5)
O4—C16	1.430 (4)	C12—H12	0.9300
C1—C2	1.340 (5)	C14—H14A	0.9600
C2—C3	1.415 (5)	C14—H14C	0.9600
C2—H2	0.9300	C14—H14B	0.9600
C3—C4	1.363 (4)	C15—H15C	0.9600
C3—H3	0.9300	C15—H15A	0.9600
C4—C5	1.480 (4)	C15—H15B	0.9600
C5—C6	1.460 (5)	C16—H16B	0.9600
C6—C7	1.327 (4)	C16—H16C	0.9600
C6—H6	0.9300	C16—H16A	0.9600
			
C1—S1—C4	90.51 (18)	O4—C10—C9	125.5 (3)
C13—O2—C14	119.5 (3)	O4—C10—C11	115.8 (3)
C11—O3—C15	116.9 (3)	C9—C10—C11	118.7 (4)
C10—O4—C16	117.5 (3)	C12—C11—O3	124.6 (3)
C2—C1—S1	113.4 (3)	C12—C11—C10	120.3 (3)
C2—C1—Cl1	126.8 (3)	O3—C11—C10	115.1 (3)
S1—C1—Cl1	119.8 (2)	C11—C12—C13	120.1 (3)
C1—C2—C3	111.9 (3)	C11—C12—H12	119.9
C1—C2—H2	124.0	C13—C12—H12	119.9
C3—C2—H2	124.0	O2—C13—C12	123.6 (3)
C4—C3—C2	112.5 (3)	O2—C13—C8	115.8 (3)
C4—C3—H3	123.7	C12—C13—C8	120.6 (3)
C2—C3—H3	123.7	O2—C14—H14A	109.5
C3—C4—C5	130.2 (3)	O2—C14—H14C	109.5
C3—C4—S1	111.7 (2)	H14A—C14—H14C	109.5
C5—C4—S1	118.1 (3)	O2—C14—H14B	109.5
O1—C5—C6	122.8 (3)	H14A—C14—H14B	109.5
O1—C5—C4	120.2 (3)	H14C—C14—H14B	109.5
C6—C5—C4	116.9 (3)	O3—C15—H15C	109.5
C7—C6—C5	121.3 (3)	O3—C15—H15A	109.5
C7—C6—H6	119.4	H15C—C15—H15A	109.5
C5—C6—H6	119.4	O3—C15—H15B	109.5
C6—C7—C8	128.4 (4)	H15C—C15—H15B	109.5
C6—C7—H7	115.8	H15A—C15—H15B	109.5
C8—C7—H7	115.8	O4—C16—H16B	109.5
C9—C8—C13	118.2 (3)	O4—C16—H16C	109.5
C9—C8—C7	122.3 (3)	H16B—C16—H16C	109.5
C13—C8—C7	119.4 (3)	O4—C16—H16A	109.5
C10—C9—C8	122.1 (3)	H16B—C16—H16A	109.5
C10—C9—H9	119.0	H16C—C16—H16A	109.5
C8—C9—H9	119.0		
			
C4—S1—C1—C2	−0.1 (3)	C16—O4—C10—C9	4.0 (6)
C4—S1—C1—Cl1	179.0 (2)	C16—O4—C10—C11	−174.8 (4)
S1—C1—C2—C3	0.4 (5)	C8—C9—C10—O4	179.8 (3)
Cl1—C1—C2—C3	−178.7 (3)	C8—C9—C10—C11	−1.4 (5)
C1—C2—C3—C4	−0.5 (5)	C15—O3—C11—C12	−11.5 (5)
C2—C3—C4—C5	−177.9 (4)	C15—O3—C11—C10	167.5 (3)
C2—C3—C4—S1	0.4 (4)	O4—C10—C11—C12	−179.8 (3)
C1—S1—C4—C3	−0.2 (3)	C9—C10—C11—C12	1.3 (5)
C1—S1—C4—C5	178.3 (3)	O4—C10—C11—O3	1.1 (5)
C3—C4—C5—O1	170.7 (4)	C9—C10—C11—O3	−177.8 (3)
S1—C4—C5—O1	−7.5 (5)	O3—C11—C12—C13	179.2 (3)
C3—C4—C5—C6	−7.5 (6)	C10—C11—C12—C13	0.2 (5)
S1—C4—C5—C6	174.3 (3)	C14—O2—C13—C12	6.9 (5)
O1—C5—C6—C7	−13.6 (6)	C14—O2—C13—C8	−172.4 (3)
C4—C5—C6—C7	164.6 (3)	C11—C12—C13—O2	179.2 (3)
C5—C6—C7—C8	−177.8 (3)	C11—C12—C13—C8	−1.6 (5)
C6—C7—C8—C9	−8.4 (6)	C9—C8—C13—O2	−179.2 (3)
C6—C7—C8—C13	168.2 (4)	C7—C8—C13—O2	4.0 (5)
C13—C8—C9—C10	0.0 (5)	C9—C8—C13—C12	1.5 (5)
C7—C8—C9—C10	176.6 (3)	C7—C8—C13—C12	−175.2 (3)

### Hydrogen-bond geometry (Å, °)

**Table:**

*D*—H···*A*	*D*—H	H···*A*	*D*···*A*	*D*—H···*A*
C3—H3···O1^i^	0.93	2.71	3.476 (4)	140
C6—H6···O1^i^	0.93	2.68	3.597 (5)	169

Symmetry codes: (i) *x*, −*y*+1/2, *z*−1/2.
